# Trends in Insulin Types and Devices Used by Adults With Type 2 Diabetes in the United States, 2016 to 2020

**DOI:** 10.1001/jamanetworkopen.2021.28782

**Published:** 2021-10-12

**Authors:** Sudipa Sarkar, James Heyward, G. Caleb Alexander, Rita R. Kalyani

**Affiliations:** 1 Division of Endocrinology, Diabetes, and Metabolism, Department of Medicine, Johns Hopkins University School of Medicine, Baltimore, Maryland; 2 Center for Drug Safety and Effectiveness, Johns Hopkins Bloomberg School of Public Health, Baltimore, Maryland; 3 Division of General Internal Medicine, Johns Hopkins Medicine, Baltimore, Maryland

## Abstract

**Importance:**

Despite rising costs and public scrutiny devoted to insulin, less is known regarding recent trends in its ambulatory use in the United States.

**Objective:**

To characterize trends in ambulatory insulin use, overall and based on insulin characteristics, among adults with type 2 diabetes in the United States from January 1, 2016, through December 31, 2020.

**Design, Setting, and Participants:**

This serial cross-sectional study included patients whose data were collected in IQVIA’s National Disease and Therapeutic Index (NDTI), a 2-stage, all-payer, nationally representative audit of outpatient care. Approximately 4800 physicians each calendar quarter completed a form for 2 consecutive days regarding visits for each of their patients, including diagnoses, treatments, and demographic information. Data were collected from January 2016 through December 2020.

**Exposures:**

Ambulatory use of insulin.

**Main Outcomes and Measures:**

Nationally representative projections for ambulatory use of insulin (ie, treatment visits), overall and aggregated by insulin molecule (insulins regular, neutral protamine Hagedorn [NPH], lispro, glulisine, glargine, detemir, degludec, and aspart), delivery devices (vials/syringes or pens), therapeutic class (short-acting, rapid-acting, long-acting, intermediate-acting, and premixed insulin), insulin type (human, analog, and biosimilar), and date of approval (newer: before 2010; and older: after 2010).

**Results:**

There were 27 860 691 insulin treatment visits between 2016 and 2020. Among all patient encounters that indicated use of insulin in 2020, 1 989 154 (43.9%) were among those aged 60 to 74 years; 2 372 629 (52.4%) among men; 2 646 247 (58.4%) among White patients; 811 639 (17.9%) among Black patients; and 701 912 (15.5%) among Hispanic patients. Insulin glargine was the most frequently used insulin from 2016 to 2020, accounting for approximately half of treatment visits (eg, 2020: 2.6 of 4.9 million visits; 95% CI, 2.1-3.1 million). Among insulin classes, long-acting insulin accounted for approximately two-thirds of treatment visits during this period (eg, 2020: 3.7 million visits; 95% CI, 3.0-4.4 million). Treatment visits for insulin pens increased from 36.1% in 2016 (2.2 of 6.0 million visits; 95% CI, 1.7-2.7 million) to 58.7% in 2020 (2.9 million visits; 95% CI, 2.3-3.5 million), while use of insulin vials/syringes declined in parallel. Analog insulin use predominated and accounted for more than 80% of total treatment visits across all years (eg, 2020: 4.3 million visits; 95% CI, 3.4-5.1 million). Newer insulins were increasingly used, from 18.1% of total treatment visits in 2016 (1.1 million visits; 95% CI, 0.8-1.4 million) to 40.9% in 2020 (2.0 million visits; 95% CI, 1.5-2.5 million). The use of biosimilar insulin, which was first approved in 2015, increased from 2.6% in 2017 (0.1 of 5.3 million visits; 95% CI, 0.04-0.2 million) to 8.2% in 2020 (0.4 million visits; 95% CI, 0.2-0.6 million) of total insulin treatment visits. The total number of insulin treatment visits declined from a peak of 6.0 million visits in 2016 to a nadir of 4.9 million visits in 2020 (approximately 18% decline).

**Conclusions and Relevance:**

In this study, ambulatory insulin use in the United States during the past 5 years remained dominated by the use of insulin analogs and insulin pen delivery devices, with increasing uptake of newer products as they have been brought to market.

## Key Points

**Question:**

What are the trends in ambulatory insulin use among adults with type 2 diabetes in the United States between 2016 and 2020?

**Findings:**

In this cross-sectional study of 27.9 million ambulatory insulin treatment visits, the use of insulin analogs and insulin glargine predominated, while the use of insulin pens and newer insulins increased during the 5 year study period.

**Meaning:**

These findings suggest that even with increased costs and scrutiny for insulin products, ambulatory use remains dominated by the use of insulin analogs and insulin pen delivery devices, with persistent uptake of newer products as they are approved.

## Teaser

This cross-sectional study characterizes trends in ambulatory insulin use, overall and based on insulin characteristics, among adults with type 2 diabetes in the United States from 2016 to 2020.

## Introduction

Insulin was first discovered 100 years ago and remains a necessary treatment for many people with diabetes. Over the past few decades, many developments in both insulin molecules and delivery devices have occurred. The human insulins that are currently available (ie, isophane and regular) were first approved by the US Food and Drug Administration (FDA) in 1982, followed by the first rapid-acting analog insulin, insulin lispro, in 1996 and the first long-acting analog insulin, insulin glargine, in 2000. The first insulin pen device was approved in 1985 and offered a new modality for insulin delivery compared with the traditional use of insulin vials and syringes.^1^

Prior studies have demonstrated that the cost of insulin per patient, including out-of-pocket cost, has gradually increased over time in the United States.^2,3^ Many have speculated that this trend in insulin expenditures is due to the increasing use of analog insulin.^4^ As an example, in 2017, the estimated average cost per vial of human Novolin N insulin was $25, while the estimated average cost per vial of Lantus insulin was $178 in the United States.^5^ In patients with type 2 diabetes, minimal to no distinct advantages in using analog insulins over human insulins have been observed in the head-to-head trials that have been done for glycemic-lowering efficacy.^6,7^ Nonetheless, analog insulins may offer greater flexibility and be more convenient for patients, with less dosing frequency and hypoglycemia.^8,9,10^

However, little is known regarding recent patterns of insulin use in the United States. While prior work has evaluated trends in insulin use,^11,12^ such work has typically evaluated trends prior to 2016 or used databases limited to a single payer, and none have examined national trends in the modality of insulin delivery. One reason this is of interest is that improved insulin adherence, insulin persistence, and lower hypoglycemia rates have been observed among patients who were initially insulin naive and who used pen devices at insulin initiation compared with those who used vials.^13,14^

Understanding recent trends in the use of insulin products and devices can inform health policy and public health initiatives. Thus, we characterized trends in ambulatory use of insulin from January 1, 2016, through December 31, 2020, in the United States. To do so, we used a comprehensive and nationally representative all-payer audit of outpatient care in the United States. In addition to quantifying aggregate use, we also characterized use based on insulin molecule; delivery devices, such as vials/syringes or pens; therapeutic class; whether insulin was human, analog, or biosimilar; and whether the product was FDA approved before or after 2010.

## Methods

We used the Health National Disease and Therapeutic Index (NDTI; IQVIA) to perform our analysis. The NDTI samples approximately 4800 physician participants quarterly and provides nationally representative diagnostic and prescribing information on patients treated by office-based physicians in the United States.^15,16^ The NDTI sample consists of office-based physicians who represent a random sample selected from the master lists of the American Medical Association and the American Osteopathic Association, stratified by geographic region and specialty. As previously described,^15^ for 2 days during each calendar quarter, individual physicians are given encounter forms to complete for each patient seen in outpatient care. The physicians’ record information detailing patient diagnosis, prescribed and known over-the-counter medication therapies, and demographic information. The NDTI is an all-payer database and thus includes visits that are paid for out of pocket as well as those covered by payers including Medicare, Medicaid, and commercial insurers. Institutional review board approval and informed consent were not sought for this analysis, in accordance with 45 CFR §46. We followed the Strengthening the Reporting of Observational Studies in Epidemiology (STROBE) reporting guideline.

Using the most recently available data from January 1, 2016, to December 31, 2020, we focused on patients with type 2 diabetes and limited analyses to individuals aged 35 years or older to increase the specificity of our method of identification for individuals with type 2 diabetes, similar to prior studies.^17^ Each diagnosis is assigned a 6-digit code, similar to those used by the *International Classification of Diseases, Ninth Revision *(*ICD-9*),^17^ and was used to identify those patients with diagnostic information consistent with type 2 diabetes. We used the Anatomical Therapeutic Classification code to identify that insulin was used. The specific insulin product was then further defined by generic and brand names in NDTI. We manually inspected the drug classification to ensure that it agreed with use in clinical care.

We included both office and telephone/telemedicine patient encounters in our study. A single patient encounter could potentially generate multiple insulin treatment visits. In other words, each insulin use, for a single patient, was considered a treatment visit. All medications that contained insulin were included in the analyses, including injected and inhaled products as well as single and combination therapies (ie, premixed formulations or insulin/glucagon-like peptide 1 [GLP-1] receptor agonist fixed dose combinations).

### Statistical Analysis

We used descriptive statistics to examine national trends in the use of insulin. Our primary unit of analysis included any visit in which physicians indicated the patient had a diagnosis of diabetes and that insulin was a treatment used by the patient (treatment visit). The data collected are projected, and sample weights were applied to create estimates for the US population accounting for the stratified cluster sampling.^17^ Given the sampling method, it is possible that a patient is included in more than a single visit. We analyzed data from 2016 to 2020 and present annual trends. We calculated 95% CIs for our estimates using tables of relative standard errors that account for the stratified sampling design of each annual audit and were provided in the IQVIA database.

We focused on the following categories: insulin class by pharmacokinetic action (ie, short-acting, rapid-acting, intermediate-acting, long-acting, or premixed), insulin type (ie, human, analog, or biosimilar), insulin delivery (ie, vial/syringe or pen device), and date of approval (ie, older: before 2010; or newer: 2010 to the present), as described in the eAppendix in the Supplement. The specific insulin products included in our analyses and their approval dates by the FDA are further described in the eTable in the Supplement. For fixed-dose insulin/GLP-1 receptor agonist combination products, we treated a single fixed-dose combination product as counting toward the respective insulin product. We defined newer insulins as those approved 2010 or later because novel insulin formulations, including ultra-long and very fast-acting insulin, as well as different insulin concentrations, including U200 (200 units/mL) and U300 (300 units/mL), were introduced after this time. We also looked at the percentages of treatment visits for insulin molecule and category compared with the total number of treatment visits each year.

## Results

There were 27 860 691 insulin treatment visits between 2016 to 2020. Among all patient encounters that indicated use of insulin in 2020, 1 989 154 (43.9%) were among those aged 60 to 74 years; 2 372 629 (52.4%) among men; 2 646 247 (58.4%) among White patients; 811 639 (17.9%) among Black patients; and 701 912 (15.5%) among Hispanic patients (Table 1).

**Table 1.** Demographic Characteristics of Ambulatory Patient Encounters With Insulin Use in the US, by Year^a^

Characteristic	Visits by year, No. (%)				
	2016	2017	2018	2019	2020
Age, y					
35-39	129 223 (2.5)	105 343 (2.3)	141 127 (2.8)	131 410 (2.5)	107 655 (2.4)
40-59	1 777 269 (35.0)	1 453 561 (31.8)	1 608 356 (32.0)	1 861 650 (35.6)	1 659 554 (36.6)
60-74	2 524 427 (49.7)	2 090 922 (45.7)	2 299 931 (45.8)	2 301 648 (44.0)	1 989 154 (43.9)
75-84	502 000 (9.9)	778 733 (17.0)	833 035 (16.6)	778 059 (14.9)	665 631 (14.7)
≥85	148 264 (2.9)	145 019 (3.2)	136 684 (2.7)	163 056 (3.1)	108 371 (2.4)
Race^b^					
Asian	252 581 (5.0)	346 677 (7.6)	197 340 (3.9)	242 329 (4.6)	230 603 (5.1)
Black	893 923 (17.6)	796 337 (17.4)	985 029 (19.6)	838 074 (16.0)	811 639 (17.9)
Hispanic	541 207 (10.7)	422 629 (9.2)	706 947 (14.1)	708 866 (13.5)	701 912 (15.5)
Other^c^	63 130 (1.2)	107 205 (2.3)	69 974 (1.4)	141 744 (2.7)	139 964 (3.1)
White	3 299 415 (64.9)	2 900 730 (63.4)	3 059 843 (61.0)	3 304 810 (63.1)	2 646 247 (58.4)
Sex^d^					
Male	2 681 365 (52.8)	2 501 060 (54.7)	2 786 861 (55.5)	2 846 302 (54.4)	2 372 629 (52.4)
Female	2 373 092 (46.7)	2 072 518 (45.3)	2 232 272 (44.5)	2 389 521 (45.6)	2 157 736 (47.6)

^a^ Data collected from the IQVIA National Disease and Therapeutic Index, 2016-2020.

^b^ A total of 30 927 visits (0.6%) did not have race specified in 2016.

^c^ The category other was not further defined in the database.

^d^ A total of 26 726 visits (0.5%) did not have sex specified in 2016.

### Insulin Class

Among the insulin classes, long-acting insulin (glargine, levemir, or degludec) accounted for most of the total treatment visits every year from 2016 to 2020, with approximately 4.0 million treatment visits (95% CI, 3.3-4.8 million) in 2016 (representing 67.3% of 6.0 million total treatment visits) to 3.7 million treatment visits (95% CI, 3.0-4.4 million) in 2020 (representing 74.8% of 4.9 million total treatment visits). Rapid-acting insulin (lispro, aspart, faster-acting aspart, glulisine) accounted for the next largest share of total treatment visits, with approximately 1.3 million treatment visits (95% CI, 0.9-1.7 million) or 21.2% of total treatment visits in 2016 and approximately 0.8 million treatment visits (95% CI, 0.5-1.1 million) or 16.5% of total treatment visits in 2020. Premixed insulin had approximately 0.5 million treatment visits (95% CI, 0.3-0.6 million) that represented 7.7% of total treatment visits in 2016 and 0.3 million treatment visits (95% CI, 0.2-0.4 million) that represented 6.0% of total treatment visits in 2020. Intermediate-acting and short-acting human insulin (NPH and regular) were a very small proportion of overall treatment visits across all years, together accounting for 0.2 million treatment visits (95% CI, 0.07-0.4 million) or 3.7% of total treatment visits in 2016 and 0.1 million treatment visits (CI 0.04-0.2 million) or 2.6% of total treatment visits in 2020.

### Insulin Type

Analog insulins (glargine, levemir, degludec, lispro, aspart, glulisine, premixed analog) accounted for most total treatment visits, with 5.6 million treatment visits (95% CI, 4.7-6.5 million) in 2016, representing 92.7% of 6.0 million total treatment visits that year, and 4.3 million treatment visits (95% CI, 3.4-5.1 million) in 2020, representing 86.3% of 4.9 million total treatment visits that year (Table 2). Human insulin (NPH, regular, and premixed human) represented 7.3% of visits in 2016 (0.4 million treatment visits; 95% CI, 0.3-0.6 million) and 5.5% of visits in 2020 (0.3 million treatment visits; 95% CI, 0.1-0.4 million). In 2017, biosimilar insulins (biosimilar glargine and biosimilar lispro) were first noted in the database and accounted for 2.6% of 5.3 million total visits (0.1 million treatment visits; 95% CI, 0.04-0.2 million), and in 2020, they accounted for 8.2% of total treatment visits (0.4 million treatment visits; 95% CI, 0.2-0.6 million). The total number of insulin treatment visits declined to a low in 2020, at 4.9 million total treatment visits compared with 6.0 million total treatment visits in 2016, representing an 18% decline.

**Table 2.** Number of Insulin Treatment Visits in the US by Type of Insulin^a^

Year	Visits, No. (%)			Total insulin visits, No. (95% CI)
	Biosimilar insulin	Human insulin	Analog insulin	
2016	0	441 461 (7.3)	5 604 310 (92.7)	6 045 771 (5 077 843-7 013 699)
2017	134 502 (2.6)	317 319 (6.0)	4 806 406 (91.4)	5 258 227 (4 361 173-6 155 281)
2018	335 839 (5.9)	417 353 (7.3)	4 935 979 (86.8)	5 689 171 (4 778 335-6 600 007)
2019	450 498 (7.6)	488 683 (8.2)	4 987 401 (84.2)	5 926 582 (4 977 736-6 875 428)
2020	405 893 (8.2)	271 069 (5.5)	4 263 978 (86.3)	4 940 940 (4 098 016-5 783 864)

^a^ Data were collected from the IQVIA National Disease and Therapeutic Index, 2016-2020.

### Insulin Molecule

**Figure 1.**
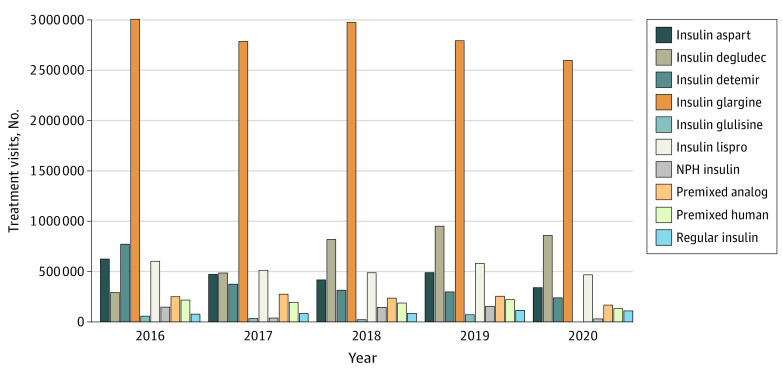
Insulin Molecules by Year, 2016 to 2020 Data source: National Disease and Therapeutic Index, 2016-2020 (IQVIA).

From 2016 to 2020, among the insulin molecules, the highest percentage of treatment visits was for insulin glargine, which consistently accounted for approximately one-half (47.1%-53.0%) of total treatment visits during the study period. The number of treatment visits for each insulin molecule by year are shown in Figure 1. In 2020, insulin glargine accounted for 52.6% of 4.9 million total treatment visits, with 2.6 million treatment visits (95% CI, 2.1-3.1 million); insulin degludec accounted for 17.4% of total treatment visits, at 0.9 million treatment visits (95% CI, 0.6-1.1 million); insulin lispro accounted for 9.5% of total treatment visits, at 0.5 million (95% CI, 0.3-0.6 million); insulin detemir accounted for 4.8% of total treatment visits, at 0.2 million treatment visits (95% CI, 0.1-0.4 million); insulin aspart accounted for 6.9% of total treatment visits, with 0.3 million treatment visits (95% CI, 0.2-0.5 million); premixed analog insulins accounted for 3.4% of total treatment visits, at 0.2 million treatment visits (95% CI, 0.08-0.3 million); premixed human insulins accounted for 2.7% of total treatment visits, at 0.1 million (95% CI, 0.04-0.2 million); regular insulin accounted for 2.2% of total treatment visits at 0.1 million treatment visits (95% CI, 0.04-0.2 million); NPH insulin accounted for 0.6% of total treatment visits, at 0.03 million treatment visits (95% CI, 0.01-0.05 million); and insulin glulisine accounted for 0% of total treatment.

### Insulin Delivery Type

**Figure 2.**
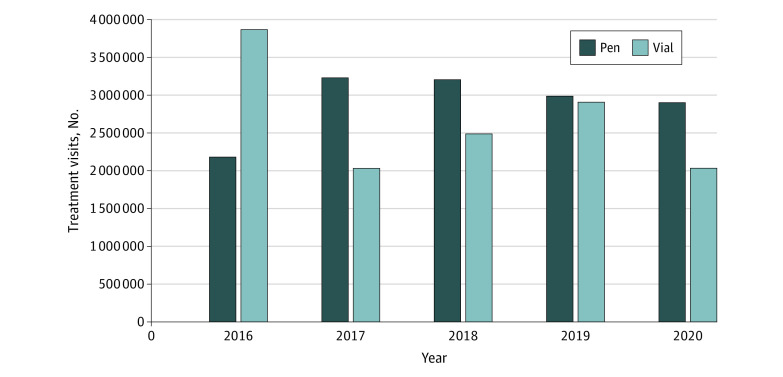
Insulin Vial/Syringe and Pen Use by Year, 2016-2020 Inhaled insulin was not included in this analysis because treatment visits accounted for less than 1% of total treatment visits during the study period. Data source: National Disease and Therapeutic Index, 2016-2020 (IQVIA).

In 2016, insulin vials/syringes accounted for 63.9% of 6.0 million total treatment visits at 3.9 million treatment visits (95% CI, 3.2-4.6 million), and insulin pens accounted for 36.1% of total treatment visits at 2.2 million treatment visits (95% CI, 1.7-2.7 million) (Figure 2). However, in 2020, insulin vials/syringes declined to 41.1% of 4.9 million total treatment visits at 2.0 million treatment visits (95% CI, 1.6-2.5 million) while insulin pens accounted for most total treatment visits, at 58.7% with 2.9 million treatment visits (95% CI, 2.3-3.5 million).

### Newer vs Older Insulins

**Figure 3.**
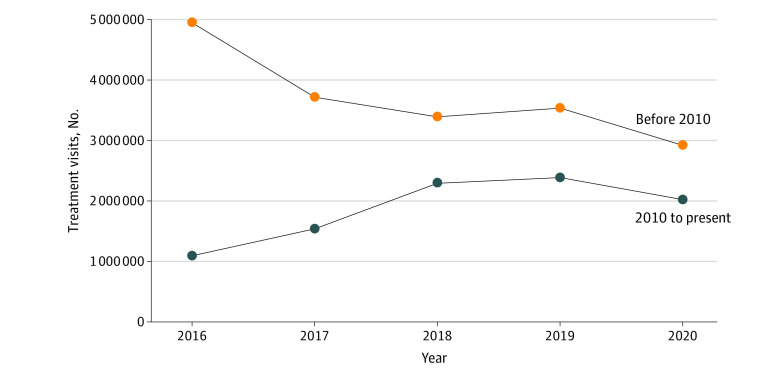
Insulin Treatment Visits for Newer vs Older Insulins, by Year, 2016-2020 Newer insulin was defined as insulins with US Food and Drug Administration approval in 2010 or later; older insulin, those with approval before 2010. Data source: National Disease and Therapeutic Index, 2016-2020 (IQVIA).

In 2016, the proportion of total patient treatment visits for older insulins was 81.9% (5.0 of 6.0 million treatment visits; 95% CI, 4.1-5.8 million), whereas the percentage of total treatment visits for newer insulins was 18.1% (1.1 million treatment visits; 95% CI, 0.8-1.4 million) (Figure 3). By 2020, the percentage of treatment visits represented by older insulins had declined to 59.1% (2.9 of 4.9 million treatment visits; 95% CI, 2.3-3.5 million), while newer insulins represented a greater percentage of treatment visits compared with 2016, at 40.9% (2.0 million treatment visits; 95% CI, 1.5-2.5 million).

## Discussion

Despite rising costs and continued scrutiny, there has been a paucity of data regarding recent trends in insulin use in the United States. In this analysis of data from a nationally representative audit of ambulatory care in the United States, we found that analog insulin accounted for more than 80% of all treatment visits, and among the insulin molecules, insulin glargine accounted for approximately half of treatment visits during the 5 year study period. In addition, the use of older insulins decreased, and the use of newer insulins steadily increased between 2016 and 2020. Interestingly, the total treatment visits for insulin was at its lowest level in 2020. These findings not only provide updated data characterizing insulin trends, but also provide greater detail regarding the use of different molecules and types of insulin, including delivery devices.^17,18^

In particular, our work extends the earlier findings of Hernandez et al,^19^ who used Medicaid claims to suggest that among that population, 80% of reimbursement for long-acting insulin units were attributed to insulin glargine (Lantus) between 2008 to 2014. However, in 2015, the market share of that drug fell to 42% in that study. This coincided with the appearance of the biosimilar insulin glargine (Basaglar) and other long-acting insulins, including insulin degludec (Tresiba) and concentrated insulin glargine (Tuojeo).^19^ We further found that insulin glargine maintained predominance 6 years after the conclusion of the Hernandez study,^19^ and we did so in this all-payer sample.

In contrast to assessments of insulin glargine, and despite their comparability with originator products,^20^ little has been published about recent trends in biosimilar insulin compared with the use of other insulins in the United States. The first biosimilar insulin to be approved in the United States was the biosimilar insulin glargine (Basaglar) in 2015. In a study of biosimilar medication use in Canada, an enormous projected potential cost savings (or unrealized savings) was observed with the use of the biosimilar (Basaglar) vs the nonbiosimilar glargine (Lantus).^21^ In addition, Hernandez et al^19^ found that the introduction of biosimilar insulin was associated with less reimbursement per milliliter of insulin overall by Medicaid, which suggested that greater competition in the insulin market was associated with lower cost of insulin. Although we did not evaluate cost in our study as other investigators have,^22^ we found that biosimilar insulin use increased by more than 200% from 2017 to 2020.

Furthermore, we noted that analog insulin accounted for most treatment visits throughout the study period. These findings are consistent with older studies using privately insured payer cohorts and reports from resource-limited countries.^2,23,24^ To our knowledge, our study is the first to document a continued predominance of analog insulin use in the United States using a multipayer database and using the most up-to-date data available through the end of 2020.

Insulin glargine’s continued predominance, relative to newer insulins, may be due to several factors, including clinical inertia, which has been commonly described in the treatment of patients with type 2 diabetes.^25^ In addition, expert guidelines note that analog basal insulins, including insulin glargine, are associated with less hypoglycemia compared with NPH (isophane) human insulin. However, they also highlight that insulin glargine U300 and insulin degludec may be associated with less hypoglycemia compared with insulin glargine U100 or insulin detemir.^26,27^

Another factor that could account for the predominance of analog insulin use may be the historic lack of available biosimilar insulins. The American Diabetes Association established a working group on the issue of insulin pricing, and in 2018, the working group published its findings, including the need for more available biosimilar insulins. It also observed that newer insulins, including analog insulins, were prescribed more often,^28^ and that prescribing human insulins for certain patients could help offset the cost of prescribing newer insulins.^29^ The 2020 American Association of Clinical Endocrinologists and the American College of Endocrinology Consensus Statement also noted that human insulins cost less than analog insulins and can be a more affordable option.^26^ Our study found that analog insulins accounted most insulin treatment visits over recent years, implying that less costly options are not being used as frequently.

Of note, we found that insulin use declined in 2020. The COVID-19 pandemic has impacted access to care and may have contributed to the reduction in insulin treatment visits and health care utilization.^30^ Prior surveillance reports of insulin use in the United States have relied on patient self-report and include younger adults, who may be more likely to have type 1 diabetes^31^; in contrast, the data for our study use information taken directly from physician encounter forms and focuses on adults with type 2 diabetes. To our knowledge, our study is the first to comprehensively report patterns of insulin use in the United States during the pandemic. Furthermore, insulin affordability and accessibility have been critically important to address, both prior to and during the pandemic. Federal and state legislations to limit insulin copay costs have been brought forward.^4^ Moreover, many pharmaceutical companies have been placing maximum caps on patient copays for insulin or offering other patient assistance programs during this time.^32^ International emergencies, such as the current pandemic, may alter the course of insulin prescribing, and further research should examine future trends.

### Strengths and Limitations

A strength of this study includes the comprehensive use of a large database that incorporates data from outpatient clinic visits across the United States. In contrast to other national databases, such as the IQVIA National Sales Perspectives database or National Prescription Audit, which focus on prescription drug expenditures and utilization in retail pharmacies, respectively, we were able to identify recent trends in the use of insulin based on patient encounters. In addition, our database included multiple payers compared with previous studies using other databases, which included single payers or only patients who had either government or private insurance.^33,34^ Moreover, we were able to include very recent data and also able to investigate trends in insulin use both by specific molecule and insulin class.

Our analysis also has limitations. First, our data do not allow for ascertainment of whether the use of insulin was appropriate according to current guidelines.^17^ Second, the NDTI provides cross-sectional data, and thus we were unable to follow individuals over time to understand dynamic components of treatment initiation or use, including adherence.^17^ Third, while the NDTI is nationally representative and generalizable to most of the US population, it does not capture health care delivery occurring in jails, prisons, or other institutional settings. Finally, because of the COVID-19 pandemic, we could not distinguish whether the decrease in treatment visits in 2020 reflects fewer overall encounters between the patient and physician or insulin use at home by patients.

## Conclusions

This cross-sectional study found important patterns of insulin use in the United States, including the predominant use of insulin glargine, the persistently greater use of analog insulins, and the increasing use of newer insulins and pen devices. An in-depth understanding of recent trends in use of insulin may provide insight into the drivers of insulin cost, although cost was not addressed in this study. Moreover, such knowledge could inform health care policy as well as guidelines on diabetes management. These findings are of broad relevance to patients, clinicians, and policy makers invested in improving access to and affordability of insulins in the United States.

## Notes

Supplement. **eAppendix.** List of Specific Insulin Products Included in IQVIA National Disease and Therapeutic Index, 2016-2020 **eTable.** Insulin Products and Devices Approved by the US Food and Drug Administration and Available in the United States, 2016-2020
