# Supplementary material for: Trends in Insulin Types and Devices Used by Adults With Type 2 Diabetes in the United States, 2016 to 2020
Source: JAMA Netw Open. 2021 Oct 12;4(10):e2128782. doi: 10.1001/jamanetworkopen.2021.28782 (PMC8511976; doi:10.1001/jamanetworkopen.2021.28782)
Supplement: Supplement. — eAppendix. List of Specific Insulin Products Included in IQVIA National Disease and Therapeutic Index, 2016-2020 eTable. Insulin Products and Devices Approved by the US Food and Drug Administration and Available in the United States, 2016-2020 [file jamanetwopen-e2128782-s001.pdf]

Supplemental Online Content

Sarkar S, Heyward J, Alexander GC, Kalyani RR. Trends in insulin types and devices used by adults with type 2 diabetes in the United States, 2016 to 2020. *JAMA Netw Open.* 2021;4(10):e2128782. doi:10.1001/jamanetworkopen.2021.28782

eAppendix. List of Specific Insulin Products Included in IQVIA National Disease and Therapeutic Index, 2016-2020

eTable. Insulin Products and Devices Approved by the US Food and Drug Administration and Available in the United States, 2016-2020

This supplemental material has been provided by the authors to give readers additional information about their work.

© 2021 Sarkar S et al. *JAMA Network Open.*

eAppendix. List of Specific Insulin Products Included in IQVIA National Disease and Therapeutic Index, 2016-2020

Insulin molecules

These included the following:

- insulin aspart (Fiasp, Fiasp Flextouch, Novolog, Novolog Flexpen)
- insulin degludec (Tresiba, Xultophy)
- insulin detemir (Levemir, Levemir Flextouch)
- insulin glargine (Basaglar Kwikpen, Lantus, Lantus Solostar, Semglee, Soliqua, Toujeo MaxSolostar, Toujeo Solostar)
- insulin glulisine (Apidra, Apidra Solostar)
- insulin lispro (Admelog, Admelog Solostar, Humalog, Humalog Jr Kwikpen, Humalog Kwikpen, Insulin Lispro, Insulin Lispro Kwikpen)*
- NPH insulin (Humulin N, Humulin N Kwikpen, Novolin N)
- regular insulin (Afrezza, Humulin R, Humulin R Kwikpen, Novolin R)
- pre-mixed analog insulins (Humalog Mix 75/25, Humalog Mix 50/50, Humalog Kwikpen 50/50, Humalog Kwikpen 75/25, Novolog Flexpen Mix 70/30, Novolog Mix 70/30)
- pre-mixed human insulins (Humulin 70/30, Humulin 70/30 Kwikpen, Novolin 70/30, Novolin 70/30 Flexpen).

**Lyumjev was approved in mid-2020 but there were no treatment visits reported for that year.*

Insulin treatment classes

These included the following:

- Long-acting insulins included Basaglar Kwikpen, Lantus, Lantus Solostar, Levemir, Levemir Flextouch, Semglee, Soliqua, Toujeo MaxSolostar, Toujeo Solostar, Tresiba, and Xultophy.
- Intermediate-acting insulins included Humulin N, Humulin N Kwikpen, and Novolin N.

© 2021 Sarkar S et al. *JAMA Network Open.*

- Rapid-acting insulins included the following: Admelog, Admelog Solostar, Afrezza, Apidra, Apidra Solostar, Fiasp, Fiasp Flextouch, Humalog, Humalog Jr Kwikpen, Humalog Kwikpen, Insulin Lispro, Insulin Lispro Kwikpen, Novolog, and Novolog Flexpen.
- Short-acting insulins included Humulin R, Humulin R Kwikpen, and Novolin R.
- Pre-mixed insulins included Humalog Kwikpen 50/50, Humalog Kwikpen 75/25, Humalog Mix 50/50, Humalog Mix 75/25, Humulin 70/30, Humulin 70/30 Kwikpen, Novolin 70/30, Novolin 70/30 Flexpen, Novolog Flexpen Mix 70/30, and Novolog Mix 70/30.

Insulin type

These included the following:

- Analog insulins included the following: Apidra, Apidra Solostar, Fiasp, Fiasp Flextouch, Humalog, Humalog Jr Kwikpen, Humalog Kwikpen, Humalog Kwikpen 50/50, Humalog Kwikpen 75/25, Humalog Mix 50/50, Humalog Mix 75/25, Insulin Lispro, Insulin Lispro Kwikpen, Lantus, Lantus Solostar, Levemir, Levemir Flextouch, Novolog, Novolog Flexpen, Novolog Flexpen Mix 70/30, Novolog Mix 70/30, Semglee, Soliqua, Toujeo MaxSolostar, Toujeo Solostar, Tresiba, Xultophy.
- Human insulins included the following: Afrezza, Humulin 70/30, Humulin 70/30 Kwikpen, Humulin N, Humulin N Kwikpen, Humulin R, Humulin R Kwikpen, Novolin 70/30, Novolin 70/30 FlexPen, Novolin N, and Novolin R.
- Biosimilar insulins included the following: Basaglar Kwikpen, Admelog, and Admelog Solostar.

Insulin delivery devices

These included the following:

© 2021 Sarkar S et al. *JAMA Network Open.*

- Vials of insulin included the following: Admelog, Apidra, Fiasp, Humalog, Humalog Mix 50/50, Humalog Mix 75/25, Humulin 70/30, Humulin N, Humulin R, Insulin Lispro, Lantus, Levemir, Novolin 70/30, Novolin N, Novolin R, Novolog, Novolog Mix 70/30, Semglee, and Tresiba.
- Insulin pens included the following: Admelog Solostar, Apidra Solostar, Basaglar Kwikpen, Fiasp Flextouch, Humalog Jr Kwikpen, Humalog Kwikpen, Humalog Kwikpen 50/50, Humalog Kwikpen 75/25, Humulin 70/30 Kwikpen, Humulin N Kwikpen, Humulin R Kwikpen, Lantus Solostar, Levemir Flextouch, Novolog Flexpen, Novolin 70/30 Flexpen, Novolog Flexpen Mix 70/30, Semglee, Soliqua, Toujeo MaxSolostar, Toujeo Solostar, and Xultophy.
- Inhaled insulin included Afrezza.

Older versus newer insulins

These included the following:

- Newer insulins included those approved by the FDA in 2010 to the present (Admelog, Admelog Solostar, Afrezza, Basaglar Kwikpen, Fiasp, Fiasp Flextouch, Humalog Jr Kwikpen, Humulin 70/30 Kwikpen, Humulin N Kwikpen, Humulin R Kwikpen, Insulin Lispro, Insulin Lispro Kwikpen, Levemir Flextouch, Novolin 70/30 Flexpen, Semglee, Soliqua, Toujeo MaxSolostar, Toujeo Solostar, Tresiba, and Xultophy).
- Older insulins included those approved by the FDA prior to 2010 (Apidra, Apidra Solostar, Humalog, Humalog Kwikpen, Humalog Kwikpen 50/50, Humalog Kwikpen 75/25, Humalog Mix 50/50, Humalog Mix 75/25, Humulin 70/30, Humulin N, Humulin R, Lantus Solostar, Lantus, Levemir, Novolin 70/30, Novolin N, Novolin R, Novolog, Novolog Flexpen, Novolog Flexpen Mix 70/30, and Novolog Mix 70/30).

© 2021 Sarkar S et al. *JAMA Network Open.*

Fixed-dose combination insulins

Combination basal and insulin-glucagon-like related peptide agents included the following:

- Soliqua (insulin glargine and lixisenatide)
- Xultophy (insulin degludec and liraglutide).

© 2021 Sarkar S et al. *JAMA Network Open.*

eTable. Insulin Products and Devices Approved by the US Food and Drug Administration and Available in the United States, 2016-2020

Insulin Molecule (Year of Approval)	Drug Brand Names	Insulin Delivery Devices (Pens)
Isophane (NPH) insulin (1982)	Humulin N® Novolin N ReliOn®<br>Novolin N®	Humulin N Kwikpen U-100<br>Novolin N FlexPen U-100<br>Novolin N FlexPen ReliOn U-100
Regular insulin (1982)	Humulin R®<br>Novolin N ReliOn®<br>Novolin R®<br>Humulin R U-500®	Novolin R FlexPen U-100<br>Novolin R FlexPen ReliOn U-100<br>Humulin R Kwikpen U-100<br>Humulin R KwikPen U-500
70% (isophane)/30% (regular) (1989)	Humulin 70/30®<br>Novolin 70/30®	Humulin 70/30 Kwikpen U-100<br>Novolin 70/30 FlexPen U-100<br>Novolin 70/30 FlexPen ReliOn U-100
Lispro (1996)	Humalog®	Humalog Kwikpen U-100<br>Humalog Kwikpen U-200<br>Humalog Jr Kwikpen U-100
50% (lispro protamine)/ 50% (lispro) (1999)	Humalog Mix 50/50®	Humalog Kwikpen 50/50 U-100
75% (lispro protamine)/25% (lispro) (1999)	Humalog Mix 75/25®	Humalog Kwikpen 75/25 U-100
Aspart (2000)	Novolog®	Novolog Flexpen U-100
Glargine (2000)	Lantus®<br>Toujeo®	Lantus Solostar U-100<br>Toujeo Solostar U-300<br>Toujeo Max Solostar U-300
70% (aspart protamine)/ 30% (aspart) (2001)	Novolog 70/30®	Novolog Flexpen Mix 70/30 U-100
Glulisine (2004)	Apidra®	Apidra Solostar U-100

© 2021 Sarkar S et al. *JAMA Network Open.*

Detemir (2005)	Levemir®	Levemir Flextouch U-100
Inhaled regular insulin (2014)	Afrezza®	Afrezza (inhaler)
Degludec (2015)	Tresiba®	Tresiba Flextouch U-100<br>Tresiba Flextouch U-200
Biosimilar glargine (2015)	Basaglar®	Basaglar Kwikpen U-100
Insulin glargine and lixisenatide (2016)	Soliqua®	Soliqua®
Insulin degludec and liraglutide (2016)	Xultophy®	Xultophy®
Faster aspart (2017)	Fiasp®	Fiasp Flextouch U-100
Biosimilar lispro (2017)	Admelog®	Admelog Solostar U-100
Biosimilar and interchangeable glargine (2020)	Semglee®	Semglee U-100 pen

© 2021 Sarkar S et al. *JAMA Network Open.*
